# The Use of Pleural Fluid to Serum Glucose Ratio in Establishing the Diagnosis of a Not So Sweet PD-Related Hydrothorax: Case Report and Literature Review

**DOI:** 10.1155/2020/8811288

**Published:** 2020-09-07

**Authors:** Nidrit Bohra, Abigayle Sullivan, Haseeb Chaudhary, Trudy Demko

**Affiliations:** ^1^Internal Medicine Department, Reading Hospital, West Reading, PA, USA; ^2^Nephrology Department, Reading Hospital, West Reading, PA, USA

## Abstract

Hydrothorax is a well-known but rare complication of peritoneal dialysis (PD), with an average incidence of 2% mainly in cases of continuous ambulatory peritoneal dialysis (CAPD). In more than 80% of these cases, the hydrothorax is attributed to an abnormal pleuroperitoneal communication. It commonly manifests as unilateral effusion, predominantly on the right. A thoracentesis to determine pleural glucose has been a diagnostic aid well relied on, as the dextrose rich dialysate raises the pleural fluid glucose. A pleural fluid glucose to serum glucose gradient greater than 50 mg/dL is suggestive of a leak with a specificity of 100% according to some studies; however, its sensitivity is variable. Our case illustrates a diagnostic dilemma due to a relatively low pleural fluid to serum glucose gradient of 21 mg/dL that caused a delay in diagnosis. A pleural fluid to serum glucose ratio >1.0 was used as a diagnostic marker that pointed toward a peritoneal leak. For confirmation, a peritoneal scintigraphy with nuclear technetium 99 scan was performed that revealed a pleuroperitoneal fistula as the source of the recurring hydrothorax in the setting of automated peritoneal dialysis (APD). The hydrothorax completely resolved with termination of APD on follow-up as the patient was transitioned to intermittent hemodialysis (HD).

## 1. Background

Given its safety and convenience as a renal replacement therapy, peritoneal dialysis (PD) is preferred at many centers around the world. Noninfective complications related to PD that are commonly seen include outflow failure, pericatheter leak, abdominal wall herniation, catheter cuff extrusion, and intestinal perforation. PD-related hydrothorax is a rare complication mainly in cases of continuous ambulatory peritoneal dialysis (CAPD) with an incidence of 1.6% according to a study [1] in 328 patients that underwent PD where 6 developed pleuroperitoneal leak.

The pleural fluid glucose, which is a representative of glucose in the dialysate, to serum glucose gradient greater than 50 mg/dL is suggestive of a peritoneal leak causing hydrothorax with a specificity of 100% according to some studies [2]; however, its sensitivity is variable [3]. We describe a case of PD-related hydrothorax with a low pleural fluid to serum (PF-S) glucose gradient but a PF-S glucose ratio >1.0 that led to the final diagnosis.

## 2. Case Presentation

We describe a case of a 63-year-old gentleman with history of heart failure with reduced ejection fraction (HFrEF) due to ischemic cardiomyopathy, bioprosthetic aortic valve, and end-stage renal disease (ESRD) on automated peritoneal dialysis (APD) for 3 months who presented with dyspnea secondary to recurrent right-sided hydrothorax. The patient reported worsening dyspnea that correlated with the initiation of peritoneal dialysis; however, pleural fluid studies performed at prior admission had revealed a transudative effusion. The low pleural fluid to serum glucose gradient was not suggestive of a peritoneal source. The patient was not in overt heart failure and was being treated with recurrent therapeutic thoracentesis, and nightly continuous cyclic peritoneal dialysis (CCPD) for 3 hours with 4.25% dialysate in a semirecumbent position was performed.

**Figure 1:**
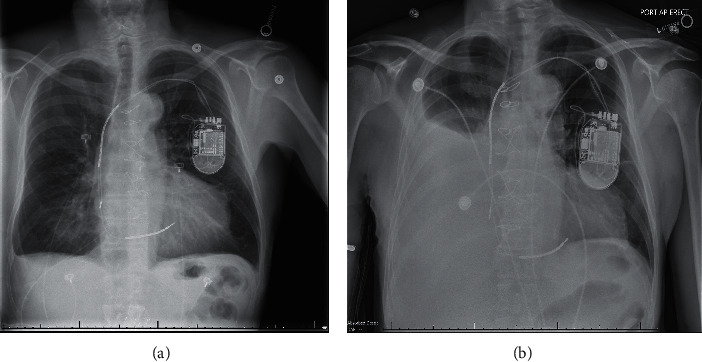
Chest radiograph illustrating worsening right pleural effusion (b) after thoracentesis (9 days prior) (a).

He presented this time with worsening dyspnea. His vitals were within normal limits. Pertinent physical exam included right-sided dull percussion note with near-absent breath sounds. The patient appeared euvolemic otherwise. Chest radiograph (CXR) showed worsening large volume right-sided hydrothorax despite a paracentesis a week prior to presentation (Figure 1). The B-type natriuretic peptide level was 172 pg/mL (0–100), and troponin I was 0.07 ng/mL (<0.06). Basic metabolic panel was significant for elevated serum creatinine (Se-Cr) 7.46 mg/dL (0.6–1.30). He initially required oxygen and had another ultrasound-guided large-volume thoracentesis, owing to his symptoms and drained 2.5 liters of clear, straw-colored fluid.

**Figure 2:**
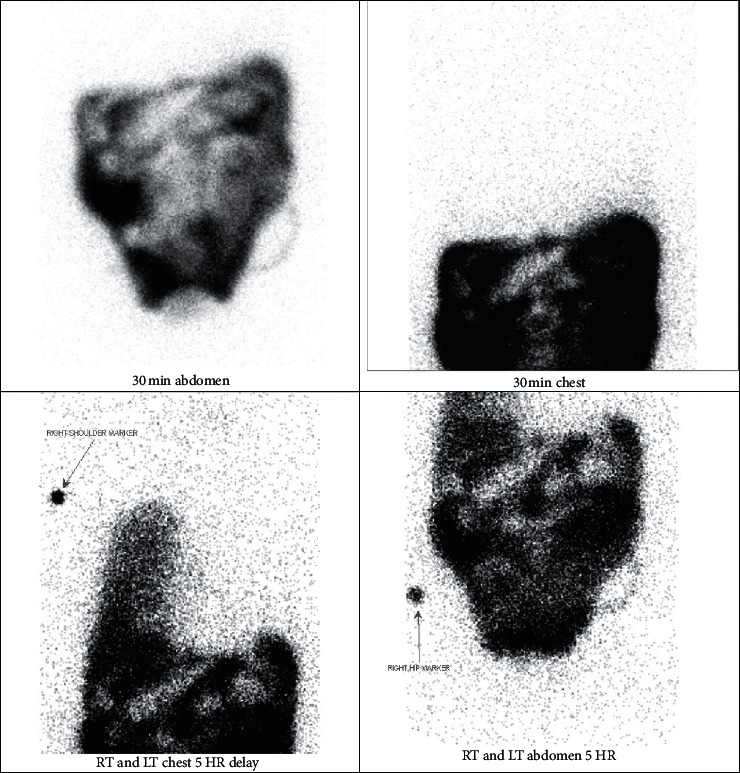
Right pleural-peritoneal fistula demonstrated on peritoneal scintigraphy after 5^th^ hour.

Repeat pleural fluid analysis was sent which revealed a transudative fluid with normal lactate dehydrogenase (76 IU/L) and protein (<3.0 g/dL) with a glucose level of 104 mg/dl (Table 1). Pleural fluid to serum glucose gradient was 21 mg/dL. Due to the high clinical index of suspicion of a peritoneal source for recurrent hydrothorax, a PF-S glucose ratio was also evaluated which was >1.0 mg/dL. In order to confirm the source, a technetium 99 peritoneal scintigraphy was performed with continuous images over a 5-hour period (Figure 2). The scan showed traversing of the isotope from the peritoneal dialysate into the pleural cavity, thus confirming the abnormal pleuroperitoneal communication.

Peritoneal dialysis was discontinued permanently with removal of the Tenckhoff catheter, and the patient was started on hemodialysis (HD). He was deemed a poor candidate for video-assisted thoracoscopic surgery (VATS) given his comorbidities and continued HD with complete resolution of the hydrothorax within 2 weeks.

## 3. Discussion

Peritoneal dialysis can be complicated by development of a hydrothorax and is most commonly seen with CAPD. Hydrothorax development is often attributed to a pleuroperitoneal fistula, an abnormal communication created between the pleural and peritoneal space that can be congenital or acquired. Acquired communication results from an abnormal increase in pressure gradient between the two spaces from an increase in intraperitoneal pressure due to the volume of dialysate [4]. Intra-abdominal pressures are higher when in a vertical, rather than supine position, and therefore predispose patients to complications including dialysate leaks. CCPD, an automated peritoneal dialysis, was developed to allow for a nightly process with larger solute and fluid removal in the supine position [5], and thus it is recommended in those at risk of complications related to increased intra-abdominal pressure gradient [4].

Initial diagnosis can be supported by increased PF-S glucose gradient, indicating presence of dialysate, but in our case, this did not prove to be a reliable indicator. There are several factors that can affect the glucose concentration in the pleural fluid coming from a peritoneal leak. It depends on the diameter of the defect causing the leakage, the rate of absorption of glucose from the pleural surface, glucose concentration of the dialysate, and a delay between the dialysate exchange and pleural fluid sample collection [6]^.^ Literature suggests that the pleural effusion is unlikely to be due to a pleuroperitoneal communication with a low PF-S glucose gradient of <50 mg/dL [7, 8]. However, there is also limited evidence that argues in favor of pleuroperitoneal communication in a PD patient as the only cause for a pleural glucose to be higher than the serum, i.e., a PF-S glucose ratio >1.0 in cases where gradient was lower than 50 mg/dl [3, 9, 10]. The PF-S glucose >1.0 in our case also supports the higher sensitivity of this approach. Pleural fluid analysis for lactate D-isomer that is only found in the dialysate is an alternate method to identify the peritoneal source [11] which was not utilized in our case.

The confirmatory tests to diagnose a peritoneal leak mainly include instillation of methylene blue, CT peritoneography, or a technetium scan, followed by serial imaging which can visualize the rate and amount of dye seepage and assess the location and size of the defect which helps in guiding management [12]. A technetium scan also has the additional advantage of being able to assess if a fistula repair has been successful, although this is often limited by cost.

First-line treatment is usually temporary halt of the PD for 2–6 weeks or switching to HD [13]. In cases where this is not attainable, conventional treatment methods involve the instillation of pleural irritants via the intercostal catheter into pleural space, such as tetracycline or talc, for pleurodesis. Surgical correction with VATS with direct pleurodesis or surgical diaphragmatic repair is more definitive with no reported recurrence but is often limited due to its invasive nature. In our case, a surgical repair was deemed high risk because of our patient's poor cardiopulmonary reserve, and he was transitioned to HD with a further plan for pleurodesis and follow-up by cardiothoracic surgery. The pleural effusion resolved with HD on follow-up.

## 4. Conclusion

PD irrespective of duration with either CAPD or APD can be complicated by a pleuroperitoneal leak. A high index of suspicion is warranted in PD patients presenting with new-onset unilateral hydrothorax when common causes are ruled out. As depicted in our case, the PF-S ratio may have higher sensitivity than PF-S gradient.

## Figures and Tables

**Table 1:** 

	Pleural fluid	Serum	PF-S gradient	PF-S ratio
Glucose (mg/dl)	104	83	21	1.2530
